# Editorial: The IL-20 Cytokines and Related Family Members in Immunity and Diseases

**DOI:** 10.3389/fimmu.2019.01976

**Published:** 2019-08-20

**Authors:** Jan Hendrik Niess, Rubén Francés

**Affiliations:** ^1^Department of Biomedicine, University of Basel, Basel, Switzerland; ^2^University Center for Gastrointestinal and Liver Diseases, St. Clara Hospital and University Hospital, Basel, Switzerland; ^3^Departamento Medicina Clínica, Universidad Miguel Hernández, Alicante, Spain; ^4^Instituto ISABIAL, Hospital General Universitario de Alicante, Alicante, Spain; ^5^CIBERehd, Instituto de Salud Carlos III, Madrid, Spain

**Keywords:** IL-20 cytokines, IL-19, IL-20, IL-24, IL-26

The IL-10 cytokine family consists of IL-10, the IL-20 subfamily which includes the cytokines IL-19, IL-20, IL-22, IL-24, and IL-26, and the IL-28 cytokine subfamily, to which the cytokines IL-28A, IL-28B, and IL-29 (also designated as type III IFNs) belong (1). In the search for genes homologous to Il10 Gallagher has cloned Il19 and Blumberg cloned Il20 15 years ago (2, 3). Jiang identified *Il24* in a melanoma cell line 20 years ago (4). However, the cytokines IL-19, IL-20, IL-24 have remained rather understudied cytokines compared to IL-22, the most prominent member of the IL-20 cytokine subfamily. Perhaps the complex structure of this cytokine family, in which individual family members have multiple pleiotropic functions and in which individual members have multiple functional receptors, makes studies with this cytokine family under considering tissue- and disease-specific contexts challenging to interpret. In general, IL-20 cytokines facilitates the communication between hematopoietic cells the interactions of hematopoietic cells with epithelial cells (5). Although multiple inflammatory diseases, such as inflammatory bowel disease, rheumatoid arthritis, in inflammatory liver diseases, vascular diseases and allergen-induced airway inflammation, lead to the expression of IL-20 cytokines specifically in affected tissues, to date, targeting strategies of individual IL-20 cytokine subfamily have not implemented clinically. Thus, this Research Topic discusses the nature of IL-20 cytokines in disease- and tissue-specific contexts.

Starting out, a review by Autieri discusses the impact of IL-19 and other members of the IL-20 cytokine family for vascular inflammatory diseases, where leucocytes, vascular smooth muscle cells, and endothelial cells express IL-19. In experimental inflammatory vascular disease, IL-19 has anti-atherosclerotic effects, is anti-stenotic, is antiproliferative, facilitates T cell differentiation to Th2 cells, and supports macrophage polarization to the “M2” phenotype. Altogether, the data summarized in the review by *Autieri* suggests that IL-19 has protective effects in vascular diseases.

The next review by Caparrós and Francés discusses the importance of IL-20 cytokines for inflammatory liver diseases, such as viral, alcoholic and non-alcoholic liver diseases, and for the progression of hepatocellular carcinoma. As summarized by *Caparrós*, the IL-20 cytokines have a wide plethora of different functions in liver diseases. For example, IL-22, on the one hand, down-regulates the release of viral proteins, but on the other, facilitates cell proliferation, recruitment of neutrophils, and induction of chemokine production. Interestingly, IL-26 supports the killing activity of NK cells during viral hepatitis. In hepatocellular carcinoma, IL-22 can promote tumor growth, whereas IL-24 inhibits the proliferation of hepatocellular carcinoma and metastasis. There is, therefore, a need to characterize IL-20 cytokines in liver diseases further to better appreciate the different functions of each in various liver diseases.

A review from our group summarizes the effects of IL-20 cytokines on intestinal diseases (Niess et al.). Although the expression of type I and II IL-20 receptor is low in the colon constitutively, recent work has indicated that IL-19 facilitates the progression of colitis. Briefly, a barrier breach and entrance of microbial-derived products induce the expression of IL-19 in macrophages. Macrophage-derived IL-19 may act on macrophages in an autocrine manner to regulate IL-6 production by this cell type. Furthermore, IL-19 could induce STAT3 phosphorylation in intestinal epithelial cells and may thereby facilitate wound healing after intestinal barrier breach.

Interestingly, Crohn's disease patients with bacterial translocation have increased serum IL-26 concentrations compared to Crohn's disease patients without bacterial translocation. Polymorphisms in *Il26* are associated with reduced bacterial killing and may thereby support recurrence of flares in Crohn's disease. Thus, the development of genetic disease models will help to further elucidate the multiple functions of individual IL-20 cytokines, before targeting individual IL-20 cytokines can be considered as a strategy to treat inflammatory bowel disease and other intestinal diseases.

The next review by Kragstrup et al. discusses different aspects of IL-20 cytokines in rheumatoid arthritis and spondyloarthritis, where IL-19 seems to have anti-inflammatory effects. In contrast, the elevated expression of IL-20 and IL-24 in the joints of patients with rheumatoid arthritis and spondyloarthritis leads to disease progression by amplifying monocyte chemoattractant protein-1 (MCP1) signaling and thereby recruiting immune cells. Phase 1 and phase 2a trials in rheumatoid arthritis tested the IL-20 antibody fletikumab with initially promising results in rheumatoid arthritis, but a phase 2b trial recently failed. Possible explanations for these disparate trial results could be the pleiotropic nature of the IL-20 cytokines and the existence of multiple receptors to which the cytokines IL-19, IL-20, IL-24 binds.

Larochette et al. review the novel properties of IL-26. IL-26 is the latest member of the IL-20 cytokine subfamily, present in humans but not mice, and its biochemical structure has cationic and amphipathic features. Thus, IL-26 is thereby not only a cytokine but also a carrier for extracellular DNA and an antimicrobial molecule. However, the nature and function of IL-26 in chronic inflammatory diseases remains to be explored. Clearly, this cytokine will be subject to further research in the upcoming years.

Finally, Weng et al. examine whether inhibiting IL-19 and its receptor (IL-20R1) protect rodents against asthma. The authors show that in Dermatophagoides pteronyssinus (Der p)-induced chronic airway inflammation, the anti-IL-20R1, and anti-IL-19 antibodies ameliorate airway hyperresponsiveness, lung immune cell infiltration, bronchial wall thickening, and Th2 cytokine expression. In addition, they report that the treatment of mice with an anti-IL-19 antibody attenuated lung inflammation in a rat ovalbumin (OVA)-induced model of asthma suggesting that inhibition of IL-20 cytokines protects from lung inflammation and that targeting IL-20 cytokines may a promising approach for the treatment of asthma.

In conclusion, the IL-20 cytokine subfamily is a rather understudied cytokine family with unique members with relevant biological activities in chronic inflammatory diseases. Since the IL-20 cytokine family is a cytokine group with high complexity, with pleiotropic natures, and existence of multiple receptors, more in-depth knowledge of individual family members in contexts of specific tissues and diseases may help to develop new strategies for the treatment of chronic inflammatory diseases.

## Author Contributions

All authors listed have made a substantial, direct and intellectual contribution to the work, and approved it for publication.

### Conflict of Interest Statement

The authors declare that the research was conducted in the absence of any commercial or financial relationships that could be construed as a potential conflict of interest.
